# Exploring Renal Malignancies in Saudi Arabia: Insights from a Tertiary Care Center Study

**DOI:** 10.15586/jkcvhl.v10i4.289

**Published:** 2023-12-18

**Authors:** Ahmed Alasker, Turki Rashed Alnafisah, Mohammad Alghafees, Belal Nedal Sabbah, Areez Shafqat, Abdullah Alhaider, Abdulrahman Alsayyari, Naif Althonayan, Mohammed AlOtaibi, Fisal Tariq Aldokhel, Salman Bin Ofisan, Saud Abdullah Alawad

**Affiliations:** 1 College of Medicine, King Saud bin Abdulaziz University for Health Sciences;; 2 Department of Urology, King Abdulaziz Medical City;; 3 Department of Medicine, King Abdullah International Medical Research Center;; 4 College of Medicine, Alfaisal University, Riyadh, Saudi Arabia;; 5 College of Medicine, Prince Sattam Bin Abdulaziz University. Al-Kharj, Saudi Arabia

**Keywords:** Kidney Cancer, Renal Cell Carcinoma, Clear Cell RCC, Chromophobe RCC, Papillary RCC

## Abstract

This retrospective study aims to describe the characteristics of renal cell carcinoma (RCC) in Saudi Arabia, in terms of epidemiology, clinical presentation, tumor subtype, Fuhrman grade, tumor size and stage, and overall survival. A total of 431 adult patients with a histopathological diagnosis of RCC between 2015 and 2023 were included in the analysis. Most patients (72.4%) had clear cell tumors, followed by chromophobe (15.1%) and papillary (12.5%) subtypes. In males, papillary RCC (85.2%) was more common compared to clear cell (59.8%) and chromophobe (67.7%) subtypes. Significant differences were observed in median body mass index (BMI) across tumor subtypes, and papillary tumor patients exhibited the highest incidence of hematuria (33.3%) compared to other subtypes. The Fuhrman grade also varied significantly among RCC types. Survival times were found to be lower for patients with papillary tumors. No significant difference was observed based on patients’ nationality. This study can inform clinical decision-making on patient prognosis and management as well as public health efforts aimed at reducing the alarming rise of RCC incidence.

## Introduction

Renal cell carcinomas (RCC) represent a heterogenous group of neoplasms arising from the nephron and are the most common forms of kidney cancer worldwide (1). A few studies have demonstrated an increasing incidence of RCC in Saudi Arabia (2, 3). The average age of RCC diagnosis in Saudi Arabia is 57 years and it predominantly affects males (~60%) (2), which is consistent with Western data (4, 5). Our group previously demonstrated that established risk factors such as smoking (25%), hypertension (53.2%), diabetes mellitus (46.2%), and obesity/overweight (73.2%) are commonly associated with RCC in Saudi Arabia and are increasing at an alarming rate in the region (2). Dyslipidemia (39.1%) was also present in a significant number of RCC patients, but its association with RCC risk and outcomes is debated (2).

The most common histologic subtypes of RCC are clear cell RCC (75–85%), papillary RCC (10–15%), and chromophobe RCC (5–10%), accounting for ~95% of RCC cases (6, 7). These subtypes are distinct in their genetic makeup and clinical behavior, manifested in differences in patterns of metastasis, recurrence, and overall survival (8–13). The regional data have identified clear cell RCC as the most prevalent subtype (2, 14), but a detailed comparison is lacking.

This retrospective study aims to describe the characteristics of RCC in Saudi Arabia in terms of its epidemiology, clinical presentation, tumor subtype, Fuhrman grade, and overall survival.

## Materials and Methods

This retrospective study included all patients aged >18 years who were diagnosed with RCC based on histopathology and received treatment between 2015 and 2023. Demographic parameters, including age, gender, body mass index, nationality, and comorbidities, were collected. Tumor-related characteristics, including histological subtype, mode of presentation, Fuhrman grade, surgical margins, tumor size, T stage, surgical and medical treatments, recurrence, metastasis, and mortality status were also recorded.

Figures were created using Microsoft Excel 2019 (Microsoft Corporation, WA, USA) and statistical analysis was performed using the Statistical Package for the Social Sciences (SPSS) version 23.0 (IBM Corporation, NY, USA). Categorical variables were presented as frequencies and percentages, while continuous variables were expressed as median and interquartile ranges (IQRs). Kaplan–Meier plots were used to depict survival curves, and differences in survival were assessed using a log-rank test. A p-value of <0.05 was considered statistically significant. The study was approved by the Institutional Review Board of King Abdullah International Medical Research Center, and patient confidentiality was ensured.

## Results

### *Demographic characteristics*

A total of 431 patients were included. A higher proportion of males had papillary RCC (85.2%) compared to clear cell (59.8%) and chromophobe (67.7%) tumors (*p* < 0.001). The proportion of Saudi patients with clear cell RCC (96.5%) was significantly higher than those with papillary (87.0%) and chromophobe (84.6%) tumors (*p* < 0.001). There was a significant difference in median BMI across the three tumor subtypes (p = 0.036). Demographic patient characteristics are recorded in Table 1.

**Table 1:** Demographic characteristics of patients by the nature of lesions.

Parameter	Category	Clear cell, N = 312	Papillary, N = 54	Chromophobe, N = 65	p-value	Missing
Gender	Male	176 (56.4%)	46 (85.2%)	44 (67.7%)	**<0.001**	0 (0%)
	Female	136 (43.6%)	8 (14.8%)	21 (32.3%)		
Age (years)	Year	59.0 (49.0, 68.5)	61.0 (54.0, 68.8)	53.0 (49.0, 64.0)	0.065	1 (0.2%)
	<45	58 (18.6%)	8 (14.8%)	12 (18.5%)	0.198	1 (0.2%)
	45–54	71 (22.8%)	7 (13.0%)	21 (32.3%)		
	55–64	76 (24.4%)	17 (31.5%)	16 (24.6%)		
	65 or more	106 (34.1%)	22 (40.7%)	16 (24.6%)		
Comorbidities	Hypertension	145 (63.9%)	28 (63.6%)	28 (65.1%)	>0.999	117 (27%)
	Diabetes	27 (11.9%)	9 (20.5%)	5 (11.6%)	0.299	117 (27%)
	Dyslipidemia	19 (8.4%)	2 (4.5%)	4 (9.3%)	0.666	117 (27%)
	Other	36 (15.9%)	5 (11.4%)	6 (14.0%)	0.816	117 (27%)
BMI (kg/m2)	kg/m2	30.7 (25.7, 35.2)	28.5 (25.7, 31.4)	29.4 (25.6, 32.9)	**0.036**	1 (0.2%)
	Underweight	8 (2.6%)	2 (3.7%)	1 (1.5%)	0.133	1 (0.2%)
	Healthy	58 (18.6%)	9 (16.7%)	10 (15.4%)		
	Overweight	72 (23.2%)	21 (38.9%)	23 (35.4%)		
	Obese	173 (55.6%)	22 (40.7%)	31 (47.7%)		
Nationality	Saudi	300 (96.5%)	47 (87.0%)	55 (84.6%)	**<0.001**	1 (0.2%)
	Non-Saudi	11 (3.5%)	7 (13.0%)	10 (15.4%)		

### *Tumor characteristics*

Hematuria was significantly more common in papillary (33.3%) than clear cell (15.5%) and chromophobe (12.5%) tumors (*p* = 0.006). Significant differences were also observed in the Fuhrman grade among clear cell, chromophobe, and papillary RCCs (p < 0.001). Tumor size and T stage at diagnosis were not significantly different between histological subtypes (p = 0.068). Regarding treatment, laparoscopic radical nephrectomy was more frequently performed for patients with clear cell carcinoma (50.3%) and chromophobe (41.5%) compared to papillary tumors (25.9%) (*p* = 0.006). Open partial nephrectomies were more commonly performed for chromophobe (16.9%) than clear cell carcinoma (5.4%) and papillary RCC (0.0%) (p < 0.001). Chemotherapy use was significantly higher among patients with papillary (11.1%) and clear cell carcinoma (7.2%) than chromophobe RCC (0.0%) (p = 0.040). Results on tumor-related parameters are presented in Table 2.

**Table 2:** Tumor characteristics by the nature of lesions.

Parameter	Category	Clear cell, N = 312	Papillary, N = 54	Chromophobe, N = 65	p-value	Missing
Mode of presentation	Loin pain	86 (27.7%)	14 (25.9%)	24 (37.5%)	0.264	3 (0.7%)
	Hematuria	48 (15.5%)	18 (33.3%)	8 (12.5%)	**0.006**	3 (0.7%)
	Incidental	173 (55.8%)	23 (42.6%)	32 (50.0%)	0.181	3 (0.7%)
Fuhrman grade	1	32 (15.8%)	8 (17.8%)	2 (13.3%)	**<0.001**	168 (39%)
	2	112 (55.2%)	11 (24.4%)	9 (60.0%)		
	3	38 (18.7%)	11 (24.4%)	4 (26.7%)		
	4	21 (10.3%)	15 (33.3%)	0 (0.0%)		
Surgical Margin	Negative	215 (80.5%)	41 (83.7%)	48 (84.2%)	0.79	58 (13%)
	Positive	52 (19.5%)	8 (16.3%)	9 (15.8%)		
Tumor Size (cm)	cm	4.0 (3.0, 7.0)	4.5 (3.5, 6.6)	5.0 (3.5, 9.0)	0.068	66 (15%)
Stage T	TX	0 (0.0%)	0 (0.0%)	0 (0.0%)	0.068	86 (20%)
	T0	0 (0.0%)	0 (0.0%)	0 (0.0%)		
	T1	126 (50.4%)	24 (55.8%)	23 (44.2%)		
	T2	11 (4.4%)	2 (4.7%)	8 (15.4%)		
	T3	113 (45.2%)	17 (39.5%)	21 (40.4%)		
Treatment offered	Open radical nephrectomy	30 (9.6%)	5 (9.3%)	5 (7.7%)	0.966	0 (0%)
	Laparoscopic radical nephrectomy	157 (50.3%)	14 (25.9%)	27 (41.5%)	**0.001**	0 (0%)
	Robotic radical nephrectomy	3 (1.0%)	3 (5.6%)	0 (0.0%)	0.053	0 (0%)
	Open partial nephrectomy	17 (5.4%)	0 (0.0%)	11 (16.9%)	**0.001**	0 (0%)
	Laparoscopic partial nephrectomy	34 (10.9%)	4 (7.4%)	4 (6.2%)	0.528	0 (0%)
	Robotic partial nephrectomy	64 (20.5%)	11 (20.4%)	16 (24.6%)	0.746	0 (0%)
	Chemotherapy	21 (7.2%)	6 (11.1%)	0 (0.0%)	**0.017**	21 (4.9%)
Local Recurrence	Yes	8 (2.8%)	2 (3.8%)	1 (1.6%)	0.796	25 (5.8%)
Metastatic Progression	Yes	31 (10.6%)	9 (16.7%)	4 (6.3%)	0.207	22 (5.1%)

### *Survival analysis*

**Figure 1:**
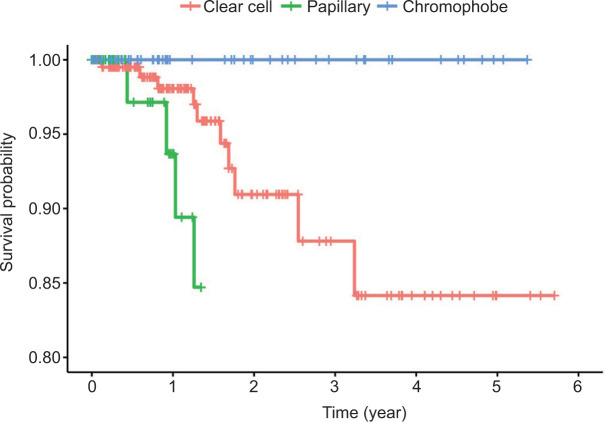
A Kaplan–Meier plot depicting survival curves across types of tumors (Chromophobe vs. Clear Cell vs. Papillary subtype).

**Figure 2:**
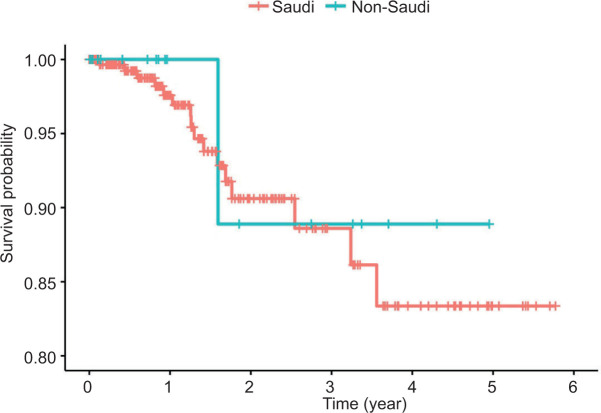
A Kaplan–Meier plot depicting overall survival curves across nationality (Saudi vs. non-Saudi).

There was a significant difference in survival across different RCC types (log-rank p = 0.003). Kaplan–Meier plots show markedly lower survival times for patients with papillary tumors (Figure 1). No significant difference was observed based on patient nationality (log-rank p = 0.750, Figure 2).

## Discussion

To the best of our knowledge, this is the largest cohort exploring RCC features in Saudi Arabia and the first to comprehensively report differences across different histologic subtypes.

Our results align with regional and international data demonstrating that the majority of RCC patients are male, clear cell histology is the most prevalent subtype, most cases are incidentally diagnosed, and most patients present with stage T1 disease (2, 9, 11, 13–15). In Saudi Arabia, an analysis of data from the Saudi Cancer Registry showed a 33% increase in documented cases from 1994 to 2006 (3). Our previous institutional study reported a 38% increase in RCC cases from 2005–2010 to 2010–2015, with 156 total cases being reported in the latter period (2). Alarmingly, the present study, spanning from 2015 to 2023, demonstrates a continuing rise in the incidence of RCC in Saudi Arabia at ~176% compared to the 2010–2015 period (431 compared to 156 cases, respectively) (2).

Our previous study observed a rise in the proportion of papillary RCC cases from 5% in 1990–1995 to 12.3% in 2010–2015, as well as the emergence of chromophobe RCC, which had no reported cases in 1990–1995 but accounted for 18.7% of all cases in 2010–2015 (2). In the present study, clear cell tumors constituted 72.4% of all RCC cases, followed by chromophobe RCC at 15.1% and papillary RCC at 12.5%. Therefore, the incidence of all three histologic subtypes is rising, but the proportions of each subtype have remained relatively stable.

At our high-volume urology center, minimally invasive surgeries (MIS) have become the preferred approach for nephrectomies, with laparoscopies and robotic techniques being increasingly utilized. Laparoscopic radical nephrectomies performed between 2000–2005 at our center accounted for 23.8% of all nephrectomies (2). The increasing use of MIS was evident in the 2010–2015 timeframe, when the majority of procedures were laparoscopic nephrectomies (53.2%) and robotic nephrectomies (1.9% of all procedures) were also introduced (2). In this study, most radical nephrectomies were conducted laparoscopically and most partial nephrectomies were done robotically. These trends indicate a trend to the predominant use of MIS for nephrectomies in tertiary care centers in Saudi Arabia.

We found histologic subtype to be a significant predictor of overall survival, but not local recurrence or metastatic progression, of RCC patients post-nephrectomy (16, 17), Contrastingly, Abu-Ghanem et al. demonstrated that patients with clear cell RCC had poorer 5-year recurrence-free survival compared to those with papillary or chromophobe RCC (13). Patterns of recurrence also varied among histological subtypes, with clear cell typically involving the lungs and bone, papillary RCC spreading to regional lymph nodes, and chromophobe RCC predominantly involving the liver and bone (13, 18). In our cohort, papillary RCC was more likely to present with higher Fuhrman grades (3 and 4) than clear cell and chromophobe RCC. However, it is important to note that the Fuhrman grade has recently shown to be inferior to the WHO/ISUP grading system in terms of prognostic predictive power (19). Validation studies have also failed to demonstrate a correlation between tumor grade evaluated by the WHO/ISUP and Fuhrman grading systems and outcome for chromophobe RCC, and it is recommended that these tumors not be graded (20).

The prognostic impact of histologic RCC subtype is controversial. A large-scale study of 4063 patients by Patard et al. demonstrated that histologic subtype of RCC was not a significant prognostic variable in a multivariate analysis (7). It should be noted that most patients in this study had advanced-stage disease, which is not representative of most RCC patients. Indeed, other studies comparing the prognostic impact of histologic subtype for early-stage RCC have reported a significant impact of histologic subtype on overall survival (9, 11, 21, 22). A regional study by Mahasin et al. found that histologic subtype is not a significant predictor of outcomes, but their sample size of chromophobe and papillary RCC patients was much smaller than our study (14).

In contrast to our results, prior studies have reported poorer survival outcomes for clear cell tumors compared to chromophobe and papillary RCC, which displayed comparable survival rates (9, 11, 21, 22). We generally observed aggressive features for papillary RCC, including a higher Fuhrman grade, comparable incidence of stage T3 lesions in clear cell RCC, higher rate of systemic chemotherapy use, and higher rates of local recurrence and metastatic progression, which may explain the poorer survival rates in these patients. The lack of a more comprehensive analysis of survival outcomes in our study prevents us from further clarifying this discrepancy.

This study has several limitations. Selection and referral biases inherent to a single-center retrospective study conducted at a tertiary care center are applicable to this study. Secondly, the TNM stage, ASA score, and ECOG PS, which are widely recognized prognostic factors, were not collected in our study (7, 11). Thirdly, data on patterns of metastasis and posttreatment recurrence sites, which have been shown to differ across histologic subtypes (12, 13), were not available. Fourthly, whether and which type of systemic therapy (i.e., anti-VEGF or tyrosine kinase inhibitors) was used for advanced-stage RCC was not determined. Lastly, a multivariate analysis to evaluate if histologic subtype remained a significant predictor of survival after adjusting for confounding factors was not conducted.

## Conclusion

The insights gained from this study can inform clinical decision-making on patient prognosis and management as well as public health efforts aimed at reducing RCC-associated risk factors. Future research in this field is essential for advancing our knowledge, refining diagnostic approaches, improving RCC patient stratification in terms of prognosis, and developing effective interventions for RCC patients in Saudi Arabia and globally.
